# Pancreatic adenocarcinomas with mature blood vessels have better overall survival

**DOI:** 10.1038/s41598-018-37909-5

**Published:** 2019-02-04

**Authors:** Eriko Katsuta, Qianya Qi, Xuan Peng, Steven N. Hochwald, Li Yan, Kazuaki Takabe

**Affiliations:** 1 Department of Surgical Oncology, Roswell Park Cancer Institute, Buffalo, NY USA; 2 Department of Biostatics and Bioinformatics, Roswell Park Cancer Institute, Buffalo, NY USA; 3 Department of Surgery, University at Buffalo the State University of New York, Jacobs School of Medicine and Biomedical Sciences, Buffalo, NY USA; 4 Department of Breast Surgery and Oncology, Tokyo Medical University, Tokyo, Japan; 5 Department of Surgery, Yokohama City University, Yokohama, Japan; 6 Department of Surgery, Niigata University Graduate School of Medical and Dental Sciences, Niigata, Japan; 7 Department of Breast Surgery, Fukushima Medical University, Fukushima, Japan

## Abstract

Pancreatic ductal adenocarcinoma (PDAC) is known for its hypovascularity. Bevacizumab, an anti-angiogenic drug, added to standard chemotherapy demonstrated no improvement in outcome for PDAC. Therefore, we hypothesized that increased vascularity may be associated with improved outcomes in PDAC possibly due to better delivery of tumor specific immune cells. To test this hypothesis, PDAC patients were classified into either high or low CD31 expression groups utilizing mRNA expression from RNA-sequence data in The Cancer Genome Atlas (TCGA) pancreatic cancer cohort. High expression of CD31, which indicates presence of more vascular endothelial cells, was associated with significantly better OS (p = 0.002). Multivariate analysis demonstrated that residual tumor (R1, 2; p = 0.026) and CD31 low expression (p = 0.007) were the only independent predictors that negatively impacted OS. Vascular stability as well as immune response related pathways were significantly upregulated in the CD31 high expressing tumors. Furthermore, there were higher proportions of anti-cancer immune cells infiltration, including activated memory CD4+ T cells (p = 0.038), CD8+ T cells (p = 0.027), gamma-delta T cells (p < 0.001) as well as naïve B cells (p = 0.006), whereas lower proportions of regulatory T cell fractions (p = 0.009), which induce an immune tolerant microenvironment, in the CD31 high expressing tumors. These findings imply that stable vessels supply anti-cancer immune cells, which are at least partially responsible for better OS in the CD31 high expressing tumors. In conclusion, CD31 high expressing PDACs have better OS, which may be due to stable vessels that supply anti-cancer immune cells.

## Introduction

Pancreatic ductal adenocarcinoma (PDAC) is the fifth major cause of cancer-related deaths in the USA^1^. In spite of the recent advances in screening, surgery, chemotherapy and radiotherapy, there has been only slight improvement in the survival of PDAC patients. According to the Surveillance, Epidemiology, and End Results (SEER) Program of the National Cancer Institute, the expected incidence of PDAC in 2018 is 55,440 cases with 44,330 deaths^2^. Although a number of risk factors have been identified, such as age, cigarette smoking, family history, and medical conditions including pancreatitis, and diabetes mellitus^3^, the cancer is often diagnosed at advanced stage, and the 5-year survival rate is less than 10%. Approximately 10% of patients have tumors localized to the pancreas, 30% of patients have locally advanced disease with tumors extending to adjacent organs and more than 50% of patients have metastatic lesions at the time of diagnosis^2^.

Angiogenesis, generation of new blood vessels, is one of the hallmarks of cancer and is well established to contribute to tumor progression. It is known that most tumors do not grow more than 3 mm in diameter without angiogenesis. Since tumor angiogenesis is essential for cancer progression, high vascularity is thought to be an aggravating factor in some cancers, however, it is also associated with better survival in others. There are few reports on the association of vascularity and prognosis in PDAC^4^. A phase III clinical trial demonstrated no improvement in outcome of PDAC with addition of the anti-angiogenic agent, bevacizumab, to standard chemotherapy, which implies that vascularity may not contribute to aggressiveness of PDAC^5^.

PDAC is characterized by a low micro-vascular density compared to other types of cancers^6,7^. Hypovascular attributes of PDAC are routinely utilized in the clinic for its diagnosis by imaging modalities such as CT scans^8^. Due to this hypovascular feature along with surrounding stroma, it is known that penetration of drugs into PDAC tumors are worse compared with other tumors. The presence of stromal components is thought to increase the interstitial fluid pressure, thus preventing drugs from penetrating the tissue interstitium^3^. In addition, the network of tumor stroma and extracellular matrix (ECM) proteins imposes a barrier for drug delivery. In PDAC, the epithelial cancer cells are surrounded by fibrotic stroma comprising activated fibroblasts, immune cells, blood vessels, and ECM. In contrast to other solid tumors where cancer-associated fibroblasts promote tumor growth and angiogenesis, the fibroblasts and fibrotic stroma in PDAC inhibit the formation and the function of blood vasculature, resulting in the sparse vasculature that is only partially functional and physically separated from the cancer cells. This unique microenvironment diminishes drug delivery via the perfusing blood vessels and therefore reduces the effectiveness of systemic chemotherapy that relies on functional vasculature for delivery to tumor cells^3^. Due to the ineffectiveness of antiangiogenic agents in PDAC and known hypovascularity of these tumors, we hypothesized that PDACs with relatively higher vascularity were associated with improved survival.

In this study, we analyzed the association between the expression of vascular related genes and survival in PDAC patients using The Cancer Genome Atlas (TCGA) cohort. Additionally, in order to resolve functional mechanisms for our observations, pathway and correlation analyses were performed.

## Results

### High expression of CD31 associates with better overall survival (OS) in PDAC patients

**Figure 1:**
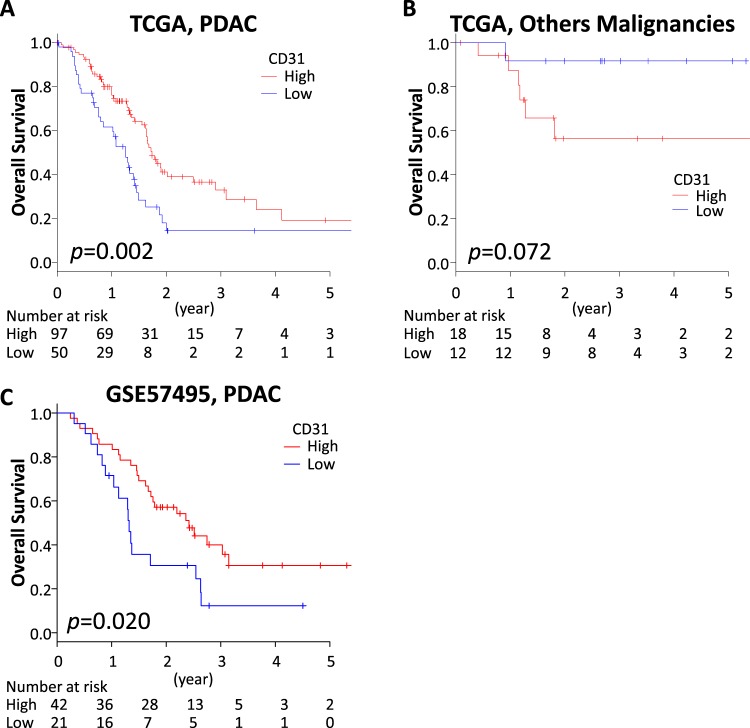
CD31 expression and patient survival. (**A**) OS in PDAC patients in TCGA pancreatic cancer cohort. (**B**) OS in pancreatic other malignancy patients in TCGA pancreatic cancer cohort. (**C**) OS in GSE57495 PDAC cohort.

**Figure 2:**
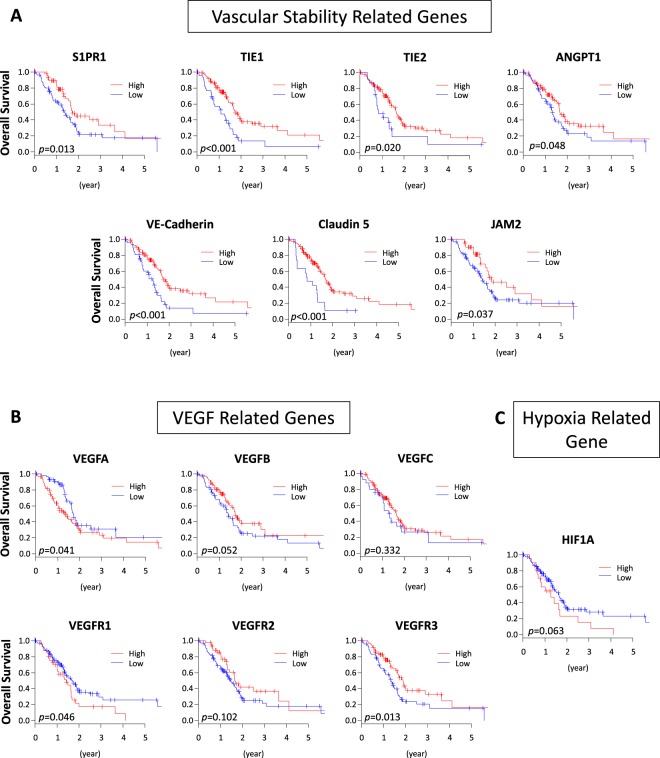
Vascular related gene expression and survival of TCGA PDAC patients. (**A**) Vascular stability related genes and (**B**) VEGF related genes. (**C**) Hypoxia related gene and patient survival.

As a marker of vascularity, we focused on CD31 (also known as PECAM1). We investigated the impact of CD31 expression on OS between PDAC and other types of pancreatic malignancies including neuroendocrine tumors that are known to be hypervascular tumors. We identified that a cutoff at lower tertile showed the highest impact on OS in the PDAC cohort, thus 100 and 50 patients were classified as high and low expression of CD31, respectively. As we expected, CD31 high expressing tumors showed significantly better OS in the PDAC cohort (Fig. 1A). Using the same cutoff point, 18 cases and 12 cases in other types of malignancies were classified into having high and low expression of CD31, respectively. Interestingly, other types of pancreatic malignancies that expressed high levels of CD31 trended to have a worse prognosis (p = 0.072) (Fig. 1B), whereas that of PDACs demonstrated a better prognosis (p = 0.002). This finding that CD31 high expressing tumors showed better prognosis in PDAC was validated using another publicly available dataset from the Gene Expression Omnibus (GEO) database (GSE57495)^9^. In this cohort, utilizing lower tertile cutoff, patients with CD31 high expressing tumors demonstrated better OS (p = 0.020) (Fig. 2).

### Low expression of CD31 is an independent prognostic factor for PDAC

Next, utilizing multivariate analysis with Cox proportional hazards regression, we investigated the influence of CD31 expression on OS compared to other factors in the PDAC cohort. Low expression of CD31 (p = 0.007) and residual tumor status (p = 0.026) were the only independent predictors of PDAC OS (Table 1).

**Table 1:** Multivariate analysis for independent OS predictors of PDAC.

Clinicopathological Factor	HR	(95% CI)	*p*
Sex (male vs female)	0.868	(0.542–1.392)	0.558
Age ( ≥ 70 vs < 70)	1.260	(0.786–2.021)	0.337
T (T3,4 vs T1,2)	0.889	(0.436–1.814)	0.747
N (N1 vs N0)	1.048	(0.584–1.883)	0.874
Histologic Grade (G3,4 vs G1,2)	1.417	(0.855–2.347)	0.176
Location (Head vs Body, Tail)	1.689	(0.778–3.669)	0.185
Residual tumor (R1,2 vs R0)	1.702	(1.065–2.719)	0.026*
CD31 (Low vs High)	1.923	(1.191–3.105)	0.007*

*p < 0.05.

### Clinicopathological demographics of CD31 high expressing PDACs

Clinicopathological demographics of PDACs were compared between CD31 high and low expressing tumors (Table 2). There was a significant greater proportion of pancreatic head tumors in the CD31 high expressing tumors compared to low expressing tumors (90% vs 24%, p = 0.027). Tumor size in the CD31 high expressing tumors was significantly smaller than in the low expressing tumors (3.6 ± 1.0 vs 4.1 ± 1.9 cm, p = 0.021). There were greater proportions of lower histological grades (Grade1 and 2) in the CD31 high expressing tumors compared to low expressing tumors (77% and 60%, p = 0.036). There were no significant differences in age, sex, residual tumor status and AJCC stages between these two groups.

**Table 2:** Clinicopathological character of CD31 high and low patients.

	CD31 high expressing tumor (n = 100)	CD31 low expressing tumor (n = 50)	*p*
Age (y.o.)^†^	63.9 ± 10.1 (40–85)	65.8 ± 12.3(35–85)	0.311
**Sex**			
Male	52(52%)	29(58%)	0.602
Female	48(48%)	21(41%)	
**Location**			
Head	89(90%)	12(24%)	0.027^*^
Body/Tail	10(10%)	37(76%)	
dTumor size (cm)^†^	3.6 ± 1.0(2.0–6.0)	4.1 ± 1.9(2.0–12.0)	0.021*
**Histological grade**			
G1/2	77(77%)	30(60%)	0.036^*^
G3/4	23(23%)	20(40%)	
**Residual tumor**			
R0	60(65%)	26(57%)	0.355
R1/2	32(35%)	20(43%)	
**AJCC Stage**			
StageI/II	95(96%)	48(96%)	>0.999
StageIII/Iv	4(4%)	2(4%)	

^†^Mean ± SD, ^*^p < 0.05.

### Vascular stability related genes are associated with better OS in PDAC

We further hypothesized that CD31 high expressing tumors demonstrated better prognosis, not due to immature fragile vessels or hypoxia, but to mature and stable vascularity. Therefore, the impact of other vascular stability related genes as well as VEGF and hypoxia related genes on the survival were analyzed. As we expected, high expression of several vascular stability related genes, including Sphingosine -1-phosphate receptor-1 (S1PR1) (p = 0.013), TIE1 (p < 0.001), TIE2 (p = 0.020), Angiopoietin 1 (ANGPT1) (p = 0.048), Vascular endothelial cadherin (VE-cadherin) (p < 0.001), Claudin 5 (p < 0.001) and Junction Adhesion Molecule 2 (JAM2) (p = 0.037), demonstrated better prognosis in the PDAC patients (Fig. 2A). High expression of some of VEGF related genes, such as VEGFA (p = 0.041) and VEGFR1 (p = 0.046), were associated with worse prognosis, while other VEGF related genes, such as VEGFB (p = 0.052), VEGFC (p = 0.332), VEGFR2 (p = 0.102), as well as hypoxia related genes, HIF1A (p = 0.063) demonstrated no significant difference between high and low expressing tumors (Fig. 2B,C). Interestingly, high expression of VEGFR3, which is the receptor of VEGFC and involved in lymphangiogenesis, was associated with a better prognosis (p = 0.013) (Fig. 2B).

**Figure 3:**
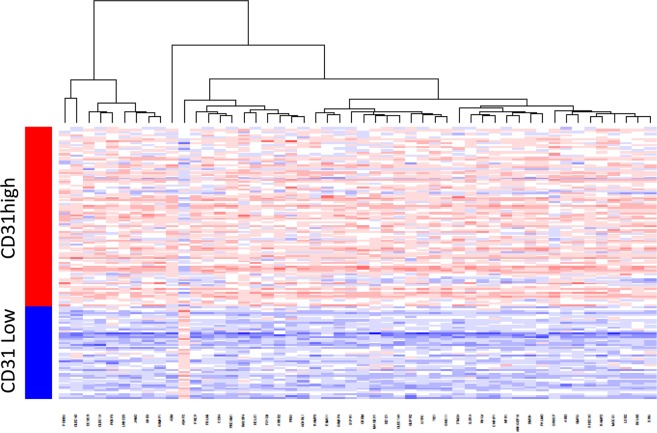
Heatmap of differently expressed genes between high and low expressing CD31 tumors among TCGA PDAC patients.

### Differential gene expression between CD31 high and low expressing PDACs

In order to explore the mechanisms as to how CD31 high expressing tumors were associated with a better prognosis in PDAC, we analyzed differently expressed genes between CD31 high and low expressing tumors. A total of 2204 genes were identified as differently expressed at Bonferroni p < 0.05. Of these genes, 1819 were upregulated and 385 were downregulated in CD31 high expressing tumors. Hierarchical clustering using the top 50 genes is shown in Fig. 3.

### CD31 expression correlates with gene expressions involved in endothelial cell markers, angiogenesis, and the immune response

**Figure 4:**
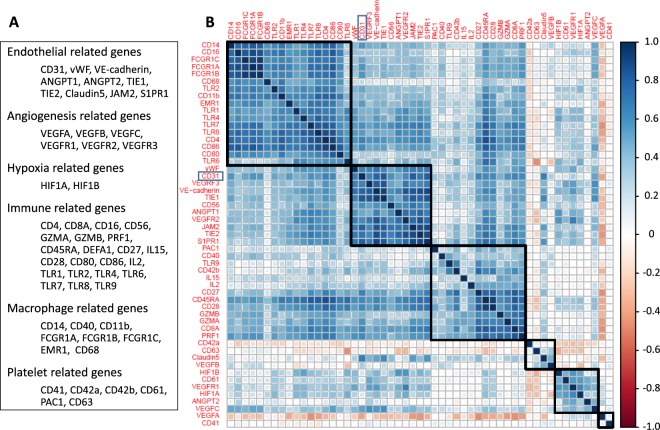
Correlation matrix of CD31 and other genes. (**A**) A list of endothelial cell markers, angiogenesis, hypoxia and immune response related genes, and macrophage and platelet surface markers. (**B**) Correlation matrix of CD31 and endothelial cell markers, angiogenesis, hypoxia and immune response related genes, and macrophage and platelet cell surface markers.

CD31 has been reported to be expressed not only in vascular endothelial cells, but also in macrophages, platelets, granulocytes and lymphocytes. In order to clarify which cells are responsible for the CD31 signature in this cohort, correlation analyses were performed. As shown in Fig. 4, CD31 expression closely correlated with almost all the other known endothelial cell marker genes; von-Willebrand factor (vWF), VE-cadherin, TIE1, TIE2, ANGPT1, JAM2 and S1PR1. CD31 also correlated with angiogenesis related genes; VEGFR2 and VEGFR3, as well as the natural killer (NK) cell marker, CD56. There was weak or no correlation with macrophage, platelet, granulocyte or lymphocyte related genes. These findings suggest that the CD31 signal that we detected mainly reflects vascular endothelial cells.

### Vascular stability and immune response related pathways are upregulated in CD31 high expressing tumors

**Figure 5:**
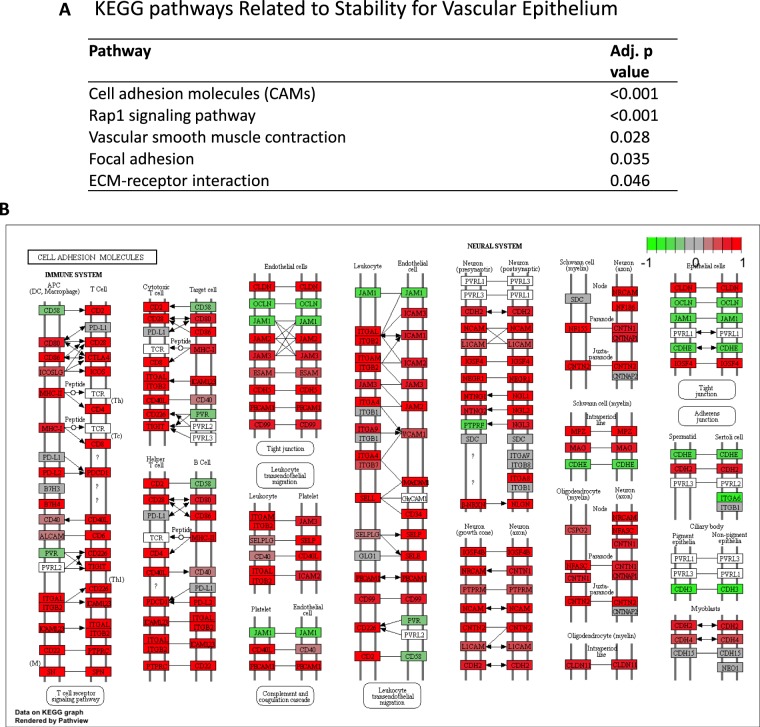
Vascular stability related pathways are upregulated in CD31 high expressing tumors. (**A**) The list of significantly upregulated vascular stability related pathways in CD31 high expressing tumors^40–42^. (**B**) Differential gene expression for “Cell adhesion molecules” pathway is demonstrated with red (high) and green (low) expression.

**Figure 6:**
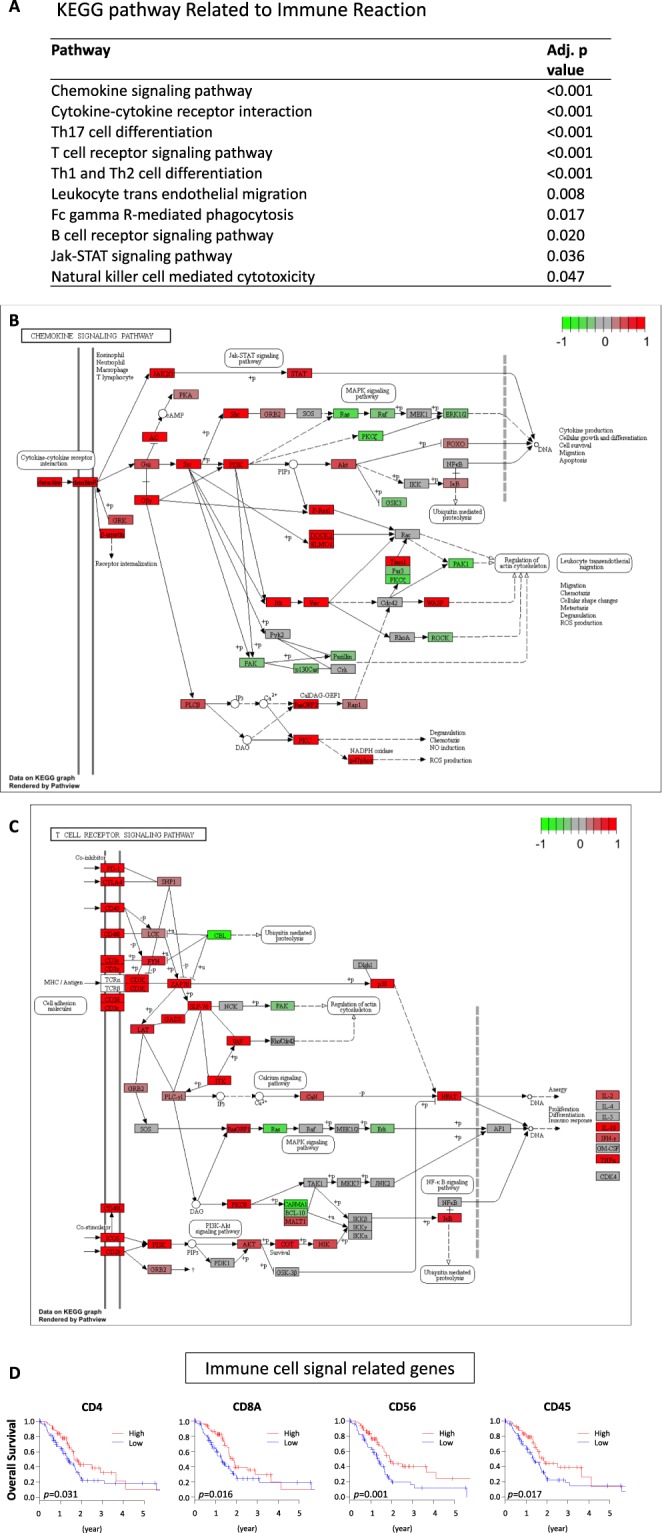
Immune reaction related pathways are upregulated in CD31 high expressing tumors. (**A**) The list of significantly upregulated immune response related pathways in CD31 high expressing tumors^40–42^. (**B**) Differential gene expression for “Chemokine signaling” and (**C**) “T cell receptor signaling” pathways is demonstrated with red (high) and green (low) expression. (**D**) Immune cell signaling related gene expression and patient survival in TCGA PDAC patients.

**Figure 7:**
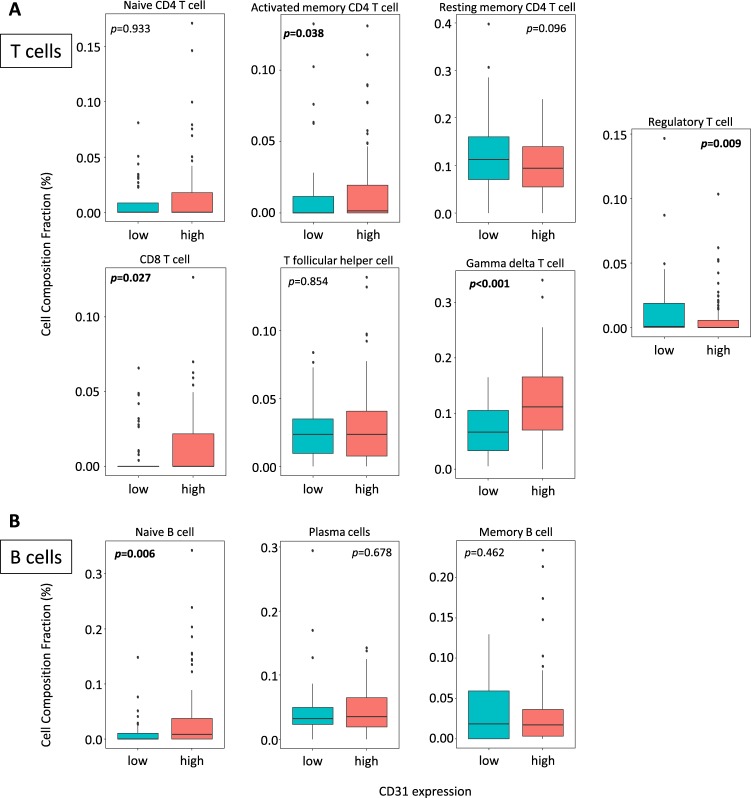
Immune cell component comparisons between CD31 high and low expressing tumors analyzed by CIBERSORT. (**A**) T cell fraction comparison. (**B**) B cell fraction comparison.

In order to obtain functional annotations of genes in our results, pathway analysis was performed. 51 pathways were identified as significantly differentially expressed between CD31 high and low expressing tumors (Supplementary Table 1). As we expected, 5 pathways were related to vascular stability, including cell adhesion molecules which showed the most significant elevations in the CD31 high tumors (Fig. 5A,B). In addition, 9 immune response related pathways, including cytokine/chemokine and lymphocyte trafficking, were upregulated in the CD31 high expressing tumors (Fig. 6A–C). This result implies that CD31 high expressing tumors have a better prognosis because of not only increased vascular stability but also a higher anti-cancer immunity. Indeed, high expression of immune response signaling related genes such as helper T cell marker CD4 (p = 0.031), cytotoxic T cell marker CD8A (p = 0.016), NK cell marker CD56 (p = 0.001), and leukocyte marker CD45 (p = 0.017) were associated with improved survival (Fig. 6D). In order to grasp the landscape of the tumor immune microenvironment, we analyzed the immune cell composition utilizing CIBERSORT algorism^10^ (Supplementary Fig. 1). We found that CD31 high expressing tumors had higher fractions of anti-cancer T cells, including CD8+ T cells (p = 0.027), activated memory CD4+ T cells (p = 0.038) and Gamma delta T cells (p < 0.001) (Fig. 7A). Interestingly, B cells including Naïve B cells, which are anti-cancer immune cells, were also accumulated in the CD31 high tumors (Fig. 7B). On the contrary, regulatory T cells (Treg), that induce immune tolerance in the tumor microenvironment, were significantly lower in those tumors (p = 0.009) (Fig. 7A).

## Discussion

In this study, we found that high expression of CD31 as well as S1PR1, TIE1, TIE2, ANGPT1, VE-Cadherin, Caludin 5 and JAM2, which are known as vascular endothelial markers, were associated with a better prognosis in PDAC patients. CD31 expression was significantly correlated with majority of vascular endothelial cell markers. CD31 high expressing tumors associated with increases in vascular stability and immune response pathways, and there were more anti-cancer immune cells in these tumors.

High vascularity is known to relate with the malignant phenotype in several types of cancers^11–14^. VEGF is a critical mediator of angiogenesis and its altered regulation is associated with several diseases, including malignancy. Bevacizumab, a recombinant humanized monoclonal antibody with a high binding specificity for VEGF, prevents its interaction with receptors on vascular endothelial cells and thereby abrogates VEGF receptor mediated intracellular signaling and resultant biologic effects^15^. For instance, high expression of VEGF and its receptor is related to worse prognosis in colon cancer^11^, thus there is no surprise that bevacizumab shows effects in these patients^15^. On the other hand, a phase III clinical trial demonstrated that the addition of bevacizumab to gemcitabine, a standard chemotherapy drug for PDAC, does not improve survival in PDAC patients^5^. This result led us to hypothesize that high expression of vascular endothelial cell related genes may be associated with a better prognosis in PDAC patients. As we expected, high expression of CD31 demonstrated better OS in PDAC patients. Interestingly, CD31 high expressing tumors demonstrated worse prognosis in other pancreatic malignant tumors, including neuroendocrine tumors, which is well known as a tumor with increased vasculature^16^. This result implies that relatively high vascularity in a typically hypovascular tumor is associated with a better prognosis, whereas that in a typically hypervascular tumor is associated with a worse prognosis.

In this study, we demonstrated strong correlation between increased CD31 expression and increased expression of vascular stability related genes as well as those pathways. Vascular stability is one of the most important factors in solid tumors^17^. VE-cadherin plays an important role as a vascular adhesion molecule, and contributes to vascular stability^18^. It has been reported that the genetic ablation of VE-cadherin in embryonic stem cells results in failure of vessel morphogenesis and is lethal^19^. CD31, Claudin5, and JAM family genes regulate adhesions of the epithelium^20–22^. This cell-cell adhesion maintains vascular maturation and stability.

On the other hand, high VEGF and VEGFR expression are essential for angiogenesis^23^. However, it is also reported that activation of VEGFR2 caused a reduction of epithelial intercellular adhesion mediated by VE-cadherin^24^. VE-cadherin regulates activation of Rho and suppresses angiogenesis mediated by VEGFR2^25^. RhoJ is an endothelial cell-restricted Rho GTPase that mediates vascular morphogenesis and is regulated by the transcription factor, ERG^26^, and is an effective and selective target for tumor angiogenesis and vascular disruption^27^. S1P receptors for the bioactive lipid S1P play an important role in vascular stability. S1PR1 and S1PR3 are expressed on vascular endothelial cells^28^. S1PR1 is critical for inhibition of angiogenesis and acquisition of vascular stability. Loss of S1PR1 leads to increased endothelial cell sprouting and the formation of ectopic vessel branches. Conversely, S1PR1 signaling inhibits angiogenic sprouting and enhances cell-to-cell adhesion. This correlates with inhibition of VEGFA induced signaling and stabilization of VE-cadherin localization at endothelial junctions^29^.

We also found that CD31 high expressing tumors were associated with upregulated immune response related pathways. Although tumor angiogenesis is critical for tumor progression, numerous studies have shown that immune cells infiltrates are observed after angiogenesis. These infiltrates involve the adaptive immune system including several types of lymphocytes as well as cells of innate immunity such as macrophages, neutrophils, eosinophils, mast cells, dendritic cells, and NK cells^30^. In addition to immune cells, surrounding stroma which consists of fibroblasts, endothelial cells, pericytes, and mesenchymal cells contribute to the tumor microenvironment. An intact immune system should be capable of eliminating neoplastic cancer cells, however, cancers inhibit the normal immune response through a variety of mechanisms, enabling malignant cells to grow and spread^31,32^; there is a dynamic relationship between a patient’s immune system and tumor cells. These diverse cells communicate with each other by means of direct contact or cytokine and chemokine production and act in autocrine and paracrine manners to control tumor growth^33^. T cell infiltration within tumors is associated with OS in patients with different cancers^34,35^. Boosting the T cells that mediate anti-cancer immune responses is an effective therapeutic tool in some types of cancers. However, T cells do not work alone. B cells drive the production of antibodies directed against tumor antigens^36,37^. Toll-like receptor (TLR) agonists mediate their anti-cancer activity through a multitude of mechanisms. High doses of TLR agonists can lead to apoptosis and have been shown to directly kill both cancer cells and ancillary cells in the tumor microenvironment. TLR activation also leads to tumor regression by increasing vascular stability and by directly or indirectly recruiting leukocytes, resulting in tumor lysis by NK and cytotoxic T cells^38^.

Although our novel findings were derived from the largest publicly available PDAC cohort, this study has limitations. The analyses of the current study were based on only the gene expression of the primary tumors in TCGA cohort. To fully explore the roles of CD31, vascular stability and immune response genes, *in vitro* and *in vivo* experimental approaches are needed.

In conclusion, tumors with increased vascular endothelial cell signals, represented by high expression of CD31, were associated with better OS in PDAC patients. It might be due to vascular stability and immune response. Further studies on the biology of PDAC should be focused on the role of CD31 in promoting vascular stability as well as immune responses.

## Methods

### TCGA Data Acquisition and Pre-Processing

Level 3 RSEM RNA-sequence data was downloaded from TCGA Data Portal and log2-transformed. Out of 185 participants in the pancreatic cancer TCGA project (PAAD), 183 cases have both OS and RNA-sequence mRNA expression data. Of these 183, 150 cases were registered as “pancreas-adenocarcinoma ductal type” in the histological type section which we analyzed as the PDAC cohort. The remaining 33 cases were registered as “pancreas-adenocarcinoma-other subtype”, “pancreas-colloid (mucinous non-cystic) carcinoma”, “pancreas-colloid (mucinous non-cystic) carcinoma” or “pancreas-neuroendocrine tumor”, which we analyzed as other types of malignancies. As a validation cohort, we obtained paired CD31 gene expression and survival profiles of 63 PDAC samples from the National Cancer for Biotechnology Information GEO database (accession codes GSE57495)^9^.

### CIBERSORT

CIBERSORT, an algorism for characterizing cell composition of complex tissues from their gene expression profiles, was used to estimate infiltrating immune cell composition using gene expression profiles^10^. Immune cell fraction data was downloaded through TCIA (https://tcia.at/home)^39^. Each immune cell fraction was compared between CD31 high and low tumors in TCGA PDAC tumors.

### Statistical analysis

OS was analyzed using Kaplan-Meier method with log-rank test, the cutoff value was determined at the point with the highest impact on survival. Patients were divided as having high or low expression of the given gene using a gene specific threshold. The Cox proportional hazards regression method was used in order to identify significant independent prognostic factors. Statistical comparisons of the clinicopathological characteristics for significance were performed by chi-square or the Fisher exact test, and a Student t-test was used to analyze the differences between continuous values. The pathway analyses were conducted using Kyoto Encyclopedia of Genes and Genomes (KEGG) pathways^40–42^ by limma package^43^, and the pathway plots were generated with PathView package^44^. In all analysis, a two-sided p < 0.05 was considered statistically significant. Pearson correlations were calculated based on the expression levels of these genes in TCGA PDAC patients and plotted. We defined that CD31, vWF, VE-cadherin, ANGPT1, ANGPT2, TIE1, TIE2, Claudin5, JAM2 and S1PR1 were “Endothelial” related genes. VEGFA, VEGFB, VEGFC, VEGFR1, VEGFR2 and VEGFR3 were “Angiogenesis” related genes. HIF1A and HIF1B were “Hypoxia” related genes. CD4, CD8A, CD16, CD56, GZMA, GZMB, PRF1, CD45RA, DEFA1, CD27, IL15, CD28, CD80, CD86, IL2, TLR1, TLR2, TLR4, TLR6, TLR7, TLR8 and TLR9 were “Immune” related genes. CD14, CD40, CD11b, FCGR1A, FCGR1B, FCGR1C, EMR1, CD68 were “Macrophage” related genes. CD41, CD42a, CD42b, CD61, PAC1 and CD63 were “Platelet” related genes. All statistical analyses were performed using R software (http://www.r-project.org/) and Bioconductor (http://bioconductor.org/).

## Supplementary information

Supplementary information
