# Supplementary material for: Pancreatic adenocarcinomas with mature blood vessels have better overall survival
Source: Sci Rep. 2019 Feb 4;9:1310. doi: 10.1038/s41598-018-37909-5 (PMC6362082; doi:10.1038/s41598-018-37909-5)
Supplement: Supplementary file 1 — Supplementary information [file 41598_2018_37909_MOESM1_ESM.pdf]

# Pancreatic adenocarcinomas with mature blood vessels have better overall survival

Eriko Katsuta[1], Qianya Qi[2], Xuan Peng[2], Steven N. Hochwald[1,3], Li Yan[2], Kazuaki Takabe[1,3-7]

[1] Department of Surgical Oncology, Roswell Park Cancer Institute, Buffalo, NY, USA
[2] Department of Biostatics and Bioinformatics, Roswell Park Cancer Institute, Buffalo, NY, USA
[3] Department of Surgery, University at Buffalo the State University of New York, Jacobs School of Medicine and Biomedical Sciences, Buffalo, NY, USA
[4] Department of Breast Surgery and Oncology, Tokyo Medical University, Tokyo, Japan
[5] Department of Surgery, Yokohama City University, Yokohama, Japan
[6] Department of Surgery, Niigata University Graduate School of Medical and Dental Sciences, Niigata, Japan
[7] Department of Breast Surgery, Fukushima Medical University, Fukushima, Japan

**Corresponding author**
Kazuaki Takabe, MD, PhD, FACS
Department of Surgical Oncology
Roswell Park Cancer Institute
Elm & Carlton Streets, Buffalo NY 14263 USA
Tel: 716-845-5540, FAX: 716-845-5705
Email: kazuaki.takabe@roswellpark.org

**Supplementary Table 1**: Significantly differentially expressed KEGG pathways between CD31 high and low tumors

| ID | Description | GeneRatio | BgRatio | pvalue | p.adjust |
|---|---|---|---|---|---|
| hsa04514 | Cell adhesion molecules (CAMs) | 64/874 | 145/7277 | 1.21E-22 | 3.66E-20 |
| hsa04640 | Hematopoietic cell lineage | 41/874 | 97/7277 | 3.98E-14 | 4.66E-12 |
| hsa05150 | Staphylococcus aureus infection | 30/874 | 56/7277 | 4.64E-14 | 4.66E-12 |
| hsa04062 | Chemokine signaling pathway | 55/874 | 185/7277 | 5.07E-11 | 3.82E-09 |
| hsa05144 | Malaria | 24/874 | 49/7277 | 1.97E-10 | 1.18E-08 |
| hsa04380 | Osteoclast differentiation | 42/874 | 128/7277 | 3.56E-10 | 1.79E-08 |
| hsa04060 | Cytokine-cytokine receptor interaction | 66/874 | 270/7277 | 5.99E-09 | 2.58E-07 |
| hsa05140 | Leishmaniasis | 28/874 | 74/7277 | 9.32E-09 | 3.50E-07 |
| hsa05323 | Rheumatoid arthritis | 29/874 | 90/7277 | 3.04E-07 | 1.02E-05 |
| hsa04659 | Th17 cell differentiation | 32/874 | 107/7277 | 5.09E-07 | 1.53E-05 |
| hsa05145 | Toxoplasmosis | 33/874 | 113/7277 | 6.21E-07 | 1.65E-05 |
| hsa04660 | T cell receptor signaling pathway | 31/874 | 103/7277 | 6.57E-07 | 1.65E-05 |
| hsa05152 | Tuberculosis | 45/874 | 179/7277 | 7.25E-07 | 1.68E-05 |
| hsa04015 | Rap1 signaling pathway | 49/874 | 210/7277 | 2.47E-06 | 5.31E-05 |
| hsa04611 | Platelet activation | 33/874 | 123/7277 | 4.94E-06 | 9.92E-05 |
| hsa05142 | Chagas disease (American trypanosomiasis) | 29/874 | 102/7277 | 5.36E-06 | 0.000101 |
| hsa04658 | Th1 and Th2 cell differentiation | 27/874 | 92/7277 | 5.86E-06 | 0.000104 |
| hsa04672 | Intestinal immune network for IgA production | 18/874 | 49/7277 | 6.91E-06 | 0.000115 |
| hsa05416 | Viral myocarditis | 20/874 | 59/7277 | 8.88E-06 | 0.000141 |
| hsa04610 | Complement and coagulation cascades | 24/874 | 79/7277 | 1.00E-05 | 0.000151 |
| hsa04670 | Leukocyte transendothelial migration | 30/874 | 112/7277 | 1.37E-05 | 0.000196 |
| hsa04064 | NF-kappa B signaling pathway | 26/874 | 95/7277 | 3.39E-05 | 0.000464 |
| hsa05330 | Allograft rejection | 14/874 | 38/7277 | 7.01E-05 | 0.000918 |
| hsa04145 | Phagosome | 35/874 | 152/7277 | 8.98E-05 | 0.001127 |
| hsa04014 | Ras signaling pathway | 47/874 | 227/7277 | 0.000104 | 0.001255 |
| hsa05310 | Asthma | 12/874 | 31/7277 | 0.000133 | 0.001541 |
| hsa05200 | Pathways in cancer | 72/874 | 395/7277 | 0.000142 | 0.001588 |
| hsa05321 | Inflammatory bowel disease (IBD) | 19/874 | 65/7277 | 0.000148 | 0.00159 |
| hsa05332 | Graft-versus-host disease | 14/874 | 41/7277 | 0.000181 | 0.001854 |
| hsa05221 | Acute myeloid leukemia | 19/874 | 66/7277 | 0.000185 | 0.001854 |
| hsa04940 | Type I diabetes mellitus | 14/874 | 43/7277 | 0.000319 | 0.003051 |
| hsa05320 | Autoimmune thyroid disease | 16/874 | 53/7277 | 0.000324 | 0.003051 |
| hsa05133 | Pertussis | 20/874 | 76/7277 | 0.000471 | 0.004296 |
| hsa05166 | HTLV-I infection | 49/874 | 255/7277 | 0.000487 | 0.004314 |
| hsa05322 | Systemic lupus erythematosus | 29/874 | 133/7277 | 0.000909 | 0.00782 |
| hsa05162 | Measles | 29/874 | 134/7277 | 0.001031 | 0.008621 |
| hsa04933 | AGE-RAGE signaling pathway in diabetic complications | 23/874 | 99/7277 | 0.001227 | 0.00998 |
| hsa04924 | Renin secretion | 17/874 | 65/7277 | 0.001322 | 0.010473 |
| hsa04666 | Fc gamma R-mediated phagocytosis | 21/874 | 91/7277 | 0.002148 | 0.01658 |
| hsa05340 | Primary immunodeficiency | 11/874 | 37/7277 | 0.003134 | 0.023014 |
| hsa04022 | cGMP-PKG signaling pathway | 32/874 | 163/7277 | 0.003135 | 0.023014 |
| hsa04662 | B cell receptor signaling pathway | 17/874 | 71/7277 | 0.003665 | 0.026265 |
| hsa04724 | Glutamatergic synapse | 24/874 | 114/7277 | 0.003947 | 0.027632 |
| hsa04921 | Oxytocin signaling pathway | 30/874 | 153/7277 | 0.004233 | 0.028027 |
| hsa05202 | Transcriptional misregulation in cancer | 35/874 | 186/7277 | 0.00426 | 0.028027 |
| hsa04270 | Vascular smooth muscle contraction | 25/874 | 121/7277 | 0.004283 | 0.028027 |
| hsa04630 | Jak-STAT signaling pathway | 30/874 | 156/7277 | 0.005668 | 0.036298 |
| hsa04510 | Focal adhesion | 36/874 | 199/7277 | 0.007259 | 0.045517 |
| hsa04512 | ECM-receptor interaction | 18/874 | 82/7277 | 0.00751 | 0.046135 |
| hsa04650 | Natural killer cell mediated cytotoxicity | 26/874 | 133/7277 | 0.007741 | 0.046601 |
| hsa04340 | Hedgehog signaling pathway | 12/874 | 47/7277 | 0.008025 | 0.047366 |

**Supplementary Figure 1**

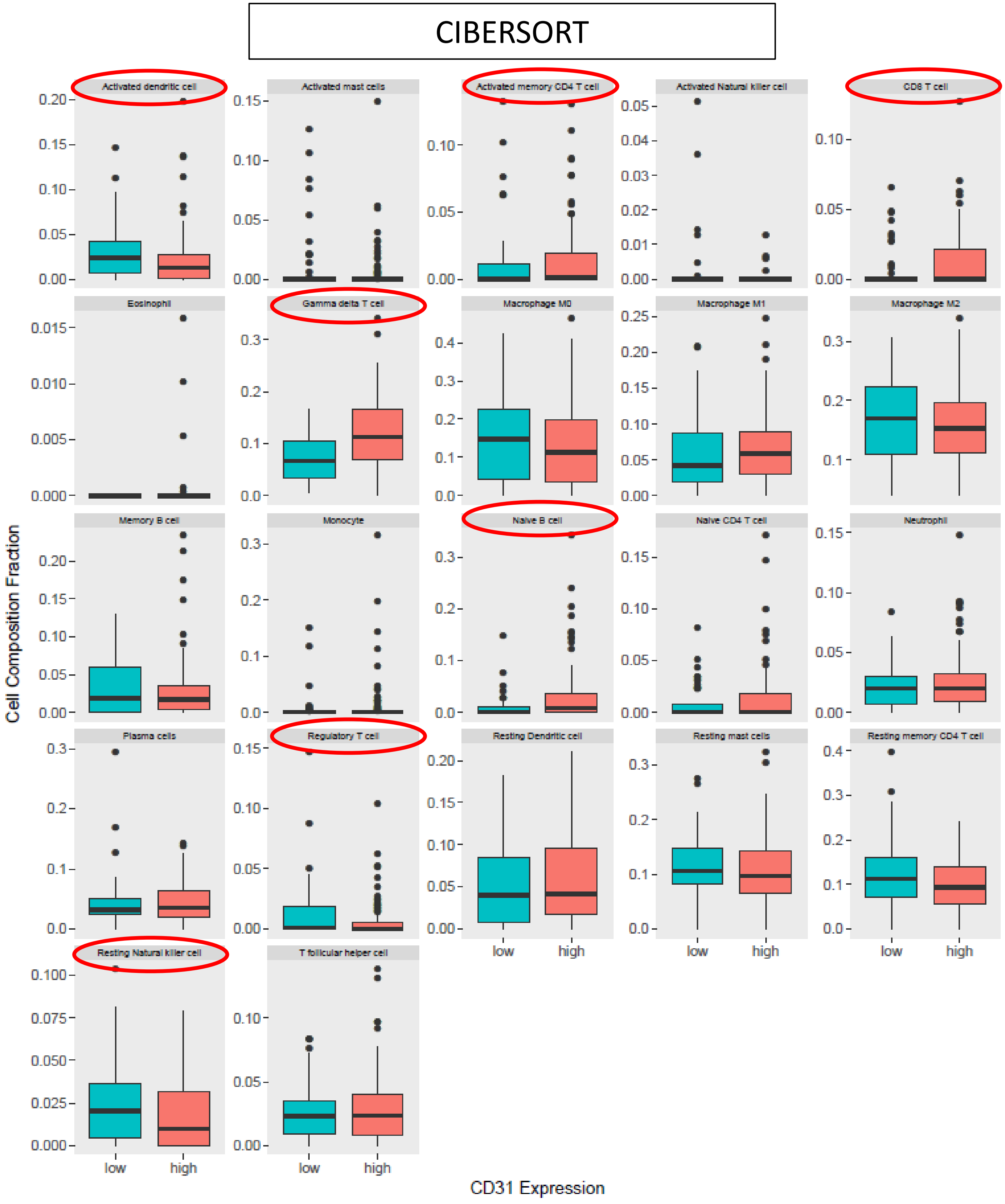


**Supplementary Figure 1.** Infiltrated immune cell comparison between CD31 high and low expressing tumors by CIBERSORT.
